# Investigation of Antigen-Antibody Interactions of Sulfonamides with a Monoclonal Antibody in a Fluorescence Polarization Immunoassay Using 3D-QSAR Models

**DOI:** 10.3390/ijms13056334

**Published:** 2012-05-23

**Authors:** Zhanhui Wang, Zhenpeng Kai, Ross C. Beier, Jianzhong Shen, Xinling Yang

**Affiliations:** 1 College of Veterinary Medicine, China Agricultural University, Beijing 100094, China; E-Mail: wangzhanhui@cau.edu.cn; 2 College of Science, China Agricultural University, Beijing 100094, China; E-Mails: zhenpengkai@hotmail.com (Z.K.); xinlingyang@cau.edu.cn (X.Y.); 3 Southern Plains Agricultural Research Center, Agricultural Research Service, U.S. Department of Agriculture, Texas A&M University, 2881 F&B Road, College Station, TX 77845-4988, USA; E-Mail: ross.beier@yahoo.com

**Keywords:** 3D-QSAR, sulfonamides, monoclonal antibody, CoMFA, CoMSIA

## Abstract

A three-dimensional quantitative structure-activity relationship (3D-QSAR) model of sulfonamide analogs binding a monoclonal antibody (MAb_SMR_) produced against sulfamerazine was carried out by Distance Comparison (DISCOtech), comparative molecular field analysis (CoMFA), and comparative molecular similarity indices analysis (CoMSIA). The affinities of the MAb_SMR_, expressed as Log_10_IC_50_, for 17 sulfonamide analogs were determined by competitive fluorescence polarization immunoassay (FPIA). The results demonstrated that the proposed pharmacophore model containing two hydrogen-bond acceptors, two hydrogen-bond donors and two hydrophobic centers characterized the structural features of the sulfonamides necessary for MAb_SMR_ binding. Removal of two outliers from the initial set of 17 sulfonamide analogs improved the predictability of the models. The 3D-QSAR models of 15 sulfonamides based on CoMFA and CoMSIA resulted in *q*^2^ *_cv_* values of 0.600 and 0.523, and *r*^2^ values of 0.995 and 0.994, respectively, which indicates that both methods have significant predictive capability. Connolly surface analysis, which mainly focused on steric force fields, was performed to complement the results from CoMFA and CoMSIA. This novel study combining FPIA with pharmacophore modeling demonstrates that multidisciplinary research is useful for investigating antigen-antibody interactions and also may provide information required for the design of new haptens.

## 1. Introduction

Sulfonamides are widely used to control a number of diseases in the animal industry and aquaculture, as well as for animal growth-promotion [1]. Despite the overall positive effects provided by sulfonamides, if inappropriate levels are used to treat livestock, fish and shrimp diseases, undesirable residues can remain in tissues, biofluids, and environmental water samples [2,3]. The presence of sulfonamide residues in foods of animal origin constitutes a potential health hazard for humans due to the increasing incidence of microbial resistance and the risk of allergic reactions to sulfonamide residues or to their metabolites.

Sulfonamides are most often detected using high-performance liquid chromatography with UV, fluorescence, or mass spectrometry detection in animal tissues, biofluids, eggs, milk, and in environmental water samples [4,5]. The availability of reproducible, sensitive and rapid methods for screening sulfonamides in foodstuffs is essential. The antibody-based analytical methods, primarily immunoassays, have proven to be useful as simple, fast and sensitive tools for detecting and quantifying sulfonamides in a variety of matrices [6].

Binding properties of a desirable antibody must meet one of two criteria to be useful in an immunoassay: an antibody must either have narrow-specificity or recognize only one analyte with high-affinity or, it must have broad-specificity and bind as many analytes as possible with similar affinity within a group of structural analogs. Several strategies in hapten design have been used while producing monoclonal antibodies (MAbs) and polyclonal antibodies against sulfonamides [7–10]. However, the affinity and specificity of the generated antibodies often have non-uniform properties. The conventional process for antibody production, when carried out without careful theoretical considerations, primarily focuses only on new hapten design and extensive screening protocols, and this approach is limited. There is considerable interest in understanding the structural basis of complex analyte-antibody interactions. A method that can provide useful information about the topological properties of a hapten can be very useful in helping produce an antibody with the desired affinity and specificity. Efforts have been made to correlate the affinity of antibodies to conformational and electronic properties of a hapten, as previously demonstrated with sulfonamides by using molecular modeling methods [8,11]. Also, reports of using a quantitative structure-activity relationship (QSAR) model for drugs and drug receptors allowed investigators to predict activity based on the structure of the involved molecules [12,13]. A three-dimensional quantitative structure-activity relationship (3D-QSAR) model is developed using comparative molecular field analysis (CoMFA) or comparative molecular similarity indices analysis (CoMSIA). The CoMFA method uses steric and electrostatic fields, whereas the CoMSIA method can use up to five physicochemical properties (steric, electrostatic, hydrophobic, and hydrogen-bond (HB) donor and HB acceptor) [14–16]. Several papers have described the use of 3D-QSAR to study binding interactions of cocaine and digoxin to MAbs by CoMFA and CoMSIA methods [17,18]. Results of the studies can be used for future redesign or selection of more suitable antibodies.

In this paper, the affinities (IC_50_ values) of a murine MAb, raised against sulfamerazine, referred to as MAb_SMR_, were determined for a set of 17 sulfonamides by fluorescence polarization immunoassay (FPIA). Structural features of the sulfonamides that demonstrated important interactions with the developed MAb_SMR_ were defined using the pharmacophore-searching program Distance Comparison (DISCOtech). An enhanced, faster version of DISCO, DISCOtech identifies features of molecular interactions that could potentially be elements of a pharmacophore model. The 3D-QSAR analysis techniques, CoMFA and CoMSIA, were used to describe the quantitative binding affinities of the sulfonamides with the MAb_SMR_. Finally, the Connolly surface of sulfamerazine (hapten), which exhibited the lowest binding affinity to the MAb_SMR_, was compared with the structures of two sulfonamide analogs. This work may develop knowledge of interactions that govern sulfonamide-antibody binding, and may help in the design of novel, performance enhanced antibodies.

## 2. Results and Discussion

### 2.1. Determination of IC_50_ Values Using FPIA

FPIA is based on the degree of movement of the observed emission intensity from the vertical to the horizontal plane, which is related to the mobility of the fluorescently labeled molecule. There is an increase in polarization of the fluorescence when a small fluorescent-labeled antigen (tracer) is bound by an antibody, and the method does not use multiple steps or separations, providing simplicity of use and higher-speed. Competitive FPIAs employing specific antibodies and fluorescein-labeled antigens for determination of drug residues in food samples have been previously studied [19,20].

**Figure 1:**
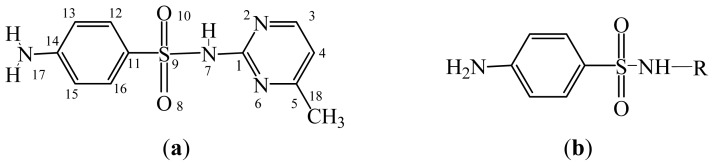
(**a**) Pharmacophore of sulfamerazine and (**b**) backbone of sulfonamides.

The MAb_SMR_ affinity for all sulfonamide analogs was expressed as IC_50_ values [21]. The structures, IC_50_ and Log_10_IC_50_ values of all sulfonamides are summarized in Table 1. The major difference among sulfonamide analogs lies in the diverse-array of R-groups linked to the nitrogen at position 7 (Figure 1a, Table 1). Simple inspection of the IC_50_ values reveals that the R-group is of primary importance for MAb_SMR_ binding of the sulfonamide analogs (Table 1). Significant differences in specificity; *i.e.*, IC_50_ values, were observed for MAb_SMR_ binding to sulfamerazine and the other 16 sulfonamide analogs. Since the MAb_SMR_ was produced to the sulfamerazine hapten, the sulfonamides with close structural similarity, such as sulfamethazine, demonstrated lower IC_50_ values than that of other analogs, which had a more diverse R-group structure from that of sulfamerazine. Even addition of a methyl group at position 3 on the pyrimidine ring, as seen in sulfamethazine, reduced the affinity compared to sulfamerazine by about 3.5-fold (Figure 1a, Table 1). A methyl group at position 5 on the pyrimidine ring resulted in favorable MAb_SMR_ binding as was illustrated by the IC_50_ value (137 ng/mL) of sulfadiazine, which does not have a methyl group at position 5 and has a reduced affinity by 7.2-fold compared to the hapten, sulfamerazine. The size influence of the groups at positions 3 and 5 on binding affinity can be observed by comparing the IC_50_ value of sulfamethazine to that of sulfadimethoxine (Table 1). It is interesting that the methoxy substituted analog, sulfameter, binds the MAb_SMR_ with a 40-fold lower affinity than does sulfamerazine. However, the effect of the methoxy oxygen atom at position 4 of the pyrimidine ring on MAb_SMR_ binding is unknown. However, the importance of the pyrimidine ring can be shown based on the affinity of the MAb_SMR_ for sulfamerazine (IC_50_ = 19 ng/mL), which is two to four orders of magnitude better than the affinity for other sulfonamide analogs where the pyrimidine ring was substituted with a different heterocyclic ring. The MAb_SMR_ has a higher-binding affinity for sulfamerazine, sulfamethazine and sulfadiazine, all of which contain a pyrimidine ring at position 7. The binding affinity of all sulfonamides spans a broad-range that exceeds four orders of magnitude and yet, except for only sulfaphenazole and sulfanilamide, most sulfonamides tested were able to significantly inhibit tracer binding to the MAb_SMR_.

It is well known that antigen-antibody binding is mainly dependent on molecular shape, defined by the geometry and low-energy interactions [22]. Inspection of the two-dimensional structures of the sulfonamide analogs could not explain the observed IC_50_ values or which forces have mainly contributed to the observed MAb_SMR_ binding to these sulfonamide analogs. In an effort to determine which structural and electronic effects were primarily important for MAb_SMR_-sulfonamide binding, studies using advanced molecular modeling techniques were undertaken. The contribution of the R-group structure to the MAb_SMR_-sulfonamide complex formation was analyzed by building pharmacophore and 3D-QSAR models using CoMFA and CoMSIA methods. Although 3D-QSAR models were initially used to direct the design of new drugs, they can also (i) elucidate the stereochemical features important for antigen binding and provide insights into the antibody binding cavity; (ii) quantitatively predict the binding affinity of antigens and antibodies; and (iii) help guide the design of a new and desirable hapten.

### 2.2. Pharmacophore and Alignment of the Sulfonamides

The pharmacophore models of all sulfonamide analogs were constructed in Sybyl 7.0 software using the DISCOtech program to perform pharmacophore elucidation from pre-computed conformations of sulfonamides that bind the MAb_SMR_. The program identifies features that can be used as elements in a pharmacophore model. The pharmacophore model defines the hydrophobic center, HB donor center and HB acceptor center. Several pharmacophore models have proven useful for identifying molecules that bind receptors and in interpreting interaction mechanisms [13,14]. The antigen-antibody complexes form as a result of several intermolecular forces; the five main forces are: (a) hydrogen-bonding; (b) coulombic, *i.e.*, electrostatic and dipole-dipole forces; (c) van der Waals; (d) hydrophobic interactions; and (e) π– π complementary ring-bonding [23]. Since adhesion forces between a small molecule and a protein are mainly non-covalent, the forces governing antigen-antibody binding are similar to that of drug-receptor binding. Finding pharmacophores for antibody recognition of antigen epitopes can be likened to finding pharmacophores for drug-receptor binding. Defining antigen epitopes and understanding antibody-binding mechanisms may lead to the development of more desirable antibodies.

**Figure 2:**
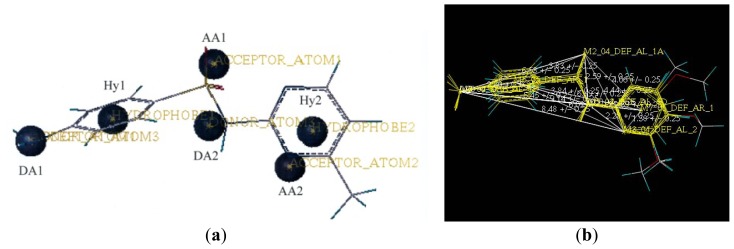
A stereoscopic view of the sulfonamide pharmacophore model derived from DISCOtech. (**a**) A stick frame representation of six sulfonamides is shown in their overlapping conformations; and (**b**) six pharmacophore feature points are shown, *i.e.*, two hydrophobic center sites (Hy1 and Hy2), two hydrogen-bond acceptor atom sites (AA1 and AA2), and two hydrogen-bond donor atom sites (DA1 and DA2).

**Figure 3:**
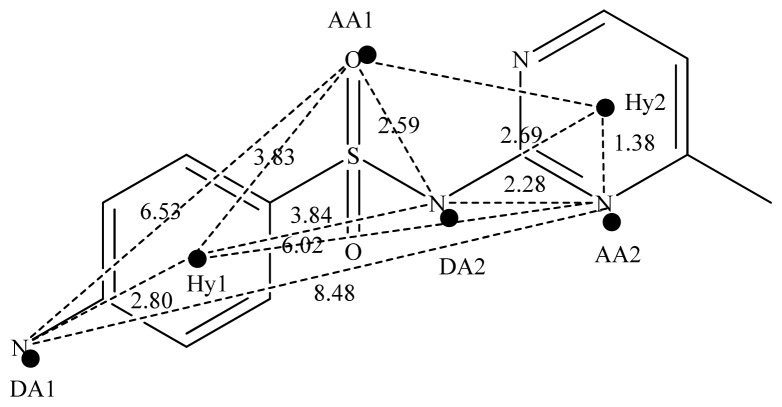
Schematic pharmacophore of the sulfonamides. Pharmacophore feature points include two hydrophobic center sites (benzene ring and pyrimidine ring), two hydrogen-bond (HB) acceptor atom sites at positions 10 and 6, and two HB donor atom sites at positions 7 and 17. They are annotated as followed: AA, HB acceptor atom; DA, HB donor atom; and Hy, hydrophobic center. The distances between these sites are shown beside each straight line in ångström units.

The DISCOtech calculation resulted in a total of 76 models, of which 11 included six sulfonamide analogs having the highest-affinity to the MAb_SMR_. The best pharmacophore model was Model 004 shown in Figure 2a having the highest score of 5.11 and greatest inter-point distance (Dmean) value of 4.60. The stick drawing shown in Figure 2b represents six sulfonamide analogs in their overlapping conformations, which includes sulfamerazine, sulfamethazine, sulfadiazine, sulfadimethoxine, sulfameter, and sulfathiazole. This pharmacophore model consists of six pharmacophore feature points; *i.e.*, two hydrophobic center sites, one on the benzene (Hy1) and the other on the heterocyclic ring (Hy2); two HB acceptor atom sites, one near the oxygen atom on the sulfonyl group at position 10 (AA1) and the other near the nitrogen at position 6 on the heterocyclic ring (AA2); and two HB donor atom sites, one on the NH_2_ group at position 17 on the benzene ring (DA1) and the other on the hydrogen-atom in the sulfonyl amine at position 7 (DA2) (Figures 1,2b). Figure 3 shows the arrangement of these features in respect to sulfamerazine. The model presented here covers all possible epitopes of the sulfonamides that bind the MAb_SMR_.

Alignment is a very important step in CoMFA studies, and some papers have shown that the resulting 3D-QSAR model is often sensitive to a particular alignment scheme [12,24]. Besides identifying the elemental characteristics required for sulfonamide binding to the MAb_SMR_, the DISCOtech derived *Model 004* also provided the alignment rule for the CoMFA and CoMSIA studies.

### 2.3. CoMFA Analysis

The general limitation of the pharmacophore model is that it does not include steric and electrostatic functionalities responsible for short and long range antigen-antibody interactions [25]. The application of CoMFA in QSAR has overcome this limitation and is intuitively helpful in understanding QSAR. Therefore, for a more detailed and rigorous analysis of sulfonamide-MAb_SMR_ interactions, the CoMFA model was used to correlate the variability in the MAb_SMR_ binding affinities to variations in sulfonamide analog molecular structure.

**Figure 4:**
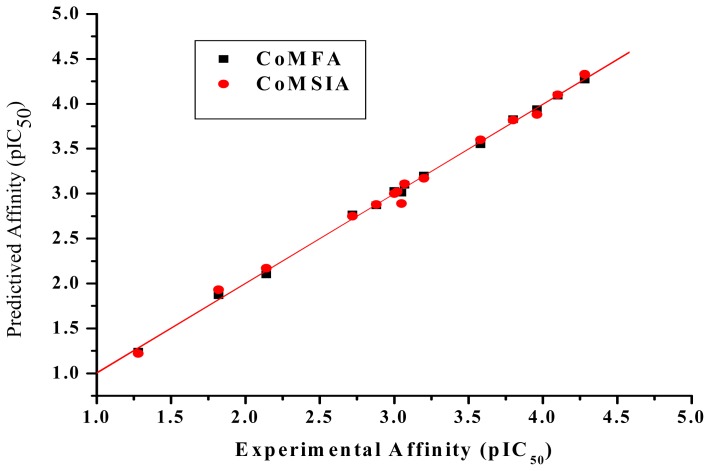
Plot of experimental versus predicted affinity values derived from the CoMFA and CoMSIA models.

Four statistically significant and chemically meaningful CoMFA models were developed based on superimposition of sulfonamide analogs. Table 2 summarizes the results of these CoMFA studies. The CoMFA *Model M1* included all 17 sulfonamide analogs, and was a poor predictive model resulting in values for leave-one-out (LOO) *q*^2^ of 0.258 and cross-validated *q*^2^ *_cv_* of 0.241. When omitting either sulfanilamide or sulfaphenazole, the *Models M2* or *M3* were obtained, respectively, and they had a better *q*^2^ *_cv_* than that of *Model M1*, but the *q*^2^ *_cv_* remained below 0.5. This may be explained because the sulfanilamide and sulfaphenazole substituent groups at position 7 are significantly different from that of sulfamerazine, the hapten used for immunization. The R-group of sulfaphenazole may add extra steric hindrance restricting binding with the MAb_SMR_; whereas, the R-group of sulfanilamide ‘H’ most likely lacks an adequate epitope to bind with the antibody. An improved CoMFA *Model M4* was obtained using 15 sulfonamide analogs, without both sulfanilamide and sulfaphenazole. *Model M4* exhibited a satisfactory predictive ability with a cross-validated *q*^2^ *_cv_* value of 0.600, non-cross-validated *r*^2^ value of 0.995 and standard error of the estimate of 0.071. The contributions of the steric and electrostatic fields to binding affinity were 55.8% and 44.2%, respectively, by PLS analysis, indicating a strong relationship between the sulfonamide analogs’ structures and binding affinities. In the case of sulfonamide-MAb_SMR_ complexes, steric interactions dominated the contribution toward the observed binding affinities. Figure 4 shows the correlation between the experimental binding affinities and the predicted ones using the CoMFA model. There was good agreement between the experimental and predicted values. Table 1 lists experimental binding affinities, predicted binding affinities and residual values (defined as the difference between experimental and predicted binding affinity) by CoMFA. The resultant residual parameters by CoMFA gave little variation between experimental and predicted binding affinities, which implied that the models were robust and would be a useful predictive tool.

**Figure 5:**
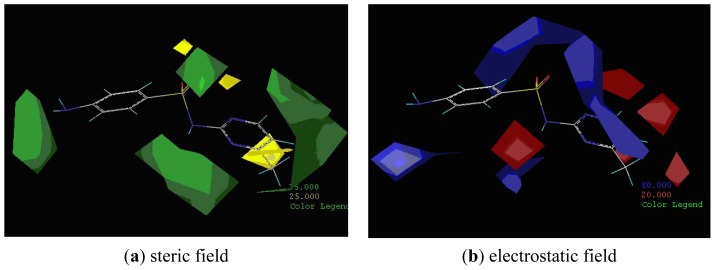
CoMFA contour plots of (**a**) steric field and (**b**) electrostatic field contributions of sulfonamides binding the MAb_SMR_.

Figure 5 features steric and electrostatic contour plots, respectively, from CoMFA analysis that show where the changes in steric and electrostatic fields are associated with MAb_SMR_ binding to the sulfonamide analogs. Greater values were correlated with more bulk near the green contours and with less bulk near the yellow contours (Figure 5a); whereas a more positive charge was correlated with the blue contours, and a more negative charge with the red contours (Figure 5b). Some of the most noticeable features in Figure 5a are the presence of large green areas that indicate improved binding affinity with increased steric tolerance in the regions near the NH_2_ group at position 17 and the NH group at position 7 of sulfamerazine (Figure 1). However, even more attention was focused on the R-group, since all sulfonamide analogs had differences in the R-group. The green steric contours from CoMFA analysis shown in Figure 5a localized near positions 3 and 4 of the pyrimidine ring indicate that bulky substituents in this region of the R-group will enhance binding affinity. However, the introduction of the methoxyl group in the pyrimidine ring at position 4 decreased the binding affinity as seen with sulfameter. This may be due to the effects of other fields like electrostatic, hydrophobic or hydrogen-bonding that may play an important role in the interactions of the sulfonamide analogs and the MAb_SMR_. In Figure 5a, the yellow-colored and green-colored regions near position 5 (the pyrimidine ring methyl), in respect to the proportion of the two colors, show that small R-groups at position 5 increase the binding affinity.

The electrostatic field of the CoMFA model is shown in Figure 5b with the structure of sulfamerazine as a reference. The large blue area above the molecule from about position 13 to position 18 represents a favorable positive Gasteiger-Hückel charge (Figure 1a). Red-colored regions near positions 3 and 5 show that suitable electronegative groups in these regions are favored to bind the MAb_SMR_. This interpretation is born out by the greater binding affinity of sulfadimethoxine, having methoxy groups at positions 3 and 5, compared with sulfameter, sulfamethoxypyridazine, or sulfachloropyridazine, which are substituted with an electronegative atom at position 4.

However, even though good *q*^2^ *_cv_* and *r*^2^ values were obtained for the CoMFA steric and electrostatic correlations with the observed MAb_SMR_ binding affinities, it was difficult to explain all structural interactions. Therefore, the more accurate 3D-QSAR technique, CoMSIA, was subsequently used to evaluate the data.

### 2.4. CoMSIA Analysis

CoMSIA is a relatively new method that adds hydrophobic, and HB donor and HB acceptor fields to the established steric and electrostatic CoMFA fields. The five fields are partitioned into spatial locations where they play a major role in determining binding affinity. Moreover, a Gaussian function is used in CoMSIA to determine the distance dependence, and the similarity indices can also be calculated at grid points inside the molecule, not just on the surface, as is the case with CoMFA.

In this study, several models were produced by using either single or multiple field combinations. The models developed using steric and electrostatic fields received a cross-validated correlation coefficient *q*^2^ *_cv_* of 0.678 (Table 3). When the hydrophobic or HB donor and HB acceptor fields were included, the *q*^2^ *_cv_* was not improved but actually decreased compared to that of the model where only steric and electrostatic fields were considered. When all five fields were included, the *q*^2^ *_cv_* decreased to 0.287. It was observed that inclusion of the hydrophobic field and HB fields did not improve the quality of the model, but produced statistically worse results. We then considered the four field model (steric, electrostatic and HB donor and HB acceptor) as the best compromise, since the *q*^2^ *_cv_* value of 0.523 and *r*^2^ value of 0.994 was acceptable, and since this model utilized a greater number of possible interactions. In the pharmacophore model, two hydrophobic centers, the benzene ring and R-group, were expected to exert an important role during formation of the antigen-antibody complex; surprisingly, the CoMSIA study revealed that the hydrophobic factor had only a negligible contribution to the sulfonamide analogs binding with the MAb_SMR_. The reason may be based on the extent of the hydrophobic interactions of the sulfonamide analogs. We speculate that the hydrophobic force may have had an effect on the interactions between the sulfonamide analogs and the MAb_SMR_, but that force may have been negligible during the process of competition compared to other forces such as steric, electrostatic, and HB donor and HB acceptor fields. The quality of the final CoMSIA model was assessed by inspection of the plots of predicted versus experimental values of Log_10_IC_50_. The CoMSIA calculated contributions of the molecular field were 10.8% steric, 39.5% electrostatic, 27.8% HB donor, and 21.9% HB acceptor.

**Figure 6:**
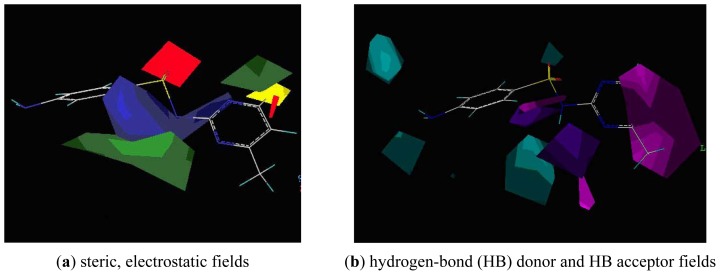
The contour plots of CoMSIA steric, electrostatic, hydrogen-bond (HB) donor and HB acceptor fields.

**Figure 7:**
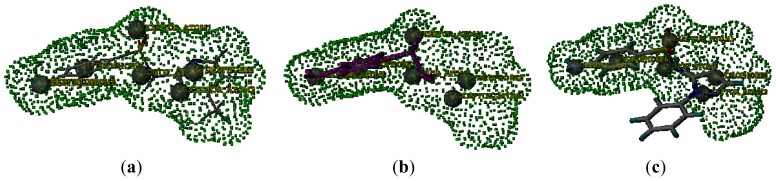
Connolly surface of (**a**) sulfamerazine expressed by the green dotted area. The optimized conformations of (**b**) sulfanilamide; and (**c**) sulfaphenazole were superimposed onto the sulfamerazine Connolly surface.

The CoMSIA contour maps are shown in Figure 6. For comparison, the molecule sulfamerazine shown in both figures had the best binding affinity. Figure 6a shows the CoMSIA contour map of the steric and electrostatic field contributions to the model. Red and blue polyhedra show regions where negatively charged and positively charged groups, respectively, will increase binding affinity. The green and yellow area located near the R-group at position 3 suggests a strict size requirement at this position. The green areas of Figure 7 represent a preferred occupancy of the MAb_SMR_ binding pocket. A variation in volume of the substituent at position 3 has a large influence on the degree of MAb_SMR_ binding with the sulfonamide analogs. Therefore, groups with increased steric bulk in these regions will enhance binding affinity. However, the yellow polyhedra indicate areas where steric bulk is unfavorable for binding. The CoMSIA contour plot (Figure 6a) appears more localized and detailed compared to the CoMFA plots (Figure 5). Based on the CoMSIA steric and electrostatic field contour map, one can speculate that spatial hindrance blocking the binding of larger steric groups is derived from amino acid residues in the MAb_SMR_ binding pocket in the area indicated by the yellow polyhedra (Figure 6a). Additionally, the importance of electrostatic interactions is shown with a red contour localized near position 3, suggesting that analogs having a heterocyclic ring with an electron-rich atom near position 3 enhance the binding affinity.

Figure 6b shows the contribution of the HB donor and HB acceptor regions of the best model derived by CoMSIA. The CoMSIA HB donor contour map shown with cyan contours near the nitrogen atom at position 17 and the oxygen atom at position 8 will be favorable for binding affinity, while the purple isopleths near the sulfonamido group at positions 7, 8, 9 and 10 suggest that a HB acceptor R-group is unfavorable for binding affinity. Magenta isopleths are mainly concentrated on the heterocyclic ring where a HB acceptor group in the sulfonamide analogs would enhance binding affinity. This is in agreement with the CoMSIA electrostatic field model, showing that an electron-rich atom acts as a HB acceptor, which explains why the compounds with a nitrogen, oxygen or sulfur atom at position 3 in the heterocyclic ring exhibit better binding than those without an electron-rich atom at this position.

### 2.5. Connolly Surface Analysis of Sulfonamides

As previously discussed in the FPIA, and CoMFA and CoMSIA sections, the MAb_SMR_ binding affinity is sensitive to an R-group with steric bulk at position 7 of the sulfonamide. In the process of deriving the CoMFA and CoMSIA models, two sulfonamide analogs, sulfaphenazole and sulfanilamide, were excluded from the data set. The QSAR derived from the CoMSIA model indicated that the steric factor plays an important role in the MAb_SMR_ binding affinity for sulfonamide analogs. Therefore, substituent size and shape clearly are important for sulfonamide binding, and this is in agreement with the generally accepted concept that shape complementarity governs antigen binding to antibodies [16,17]. In this study, the importance of shape complementarity is reflected in the low affinities displayed by sulfaphenazole and sulfanilamide. The requirement for the appropriate size steric bulk of the R-group for high-binding affinity can be seen by observing the IC_50_ values (Table 1). All sulfonamide analogs show a lower-affinity to the MAb_SMR_ than does sulfamerazine. For clarity purposes, the structures of two sulfonamide analogs, sulfaphenazole and sulfanilamide, were correlated with the Connolly surface of sulfamerazine. The Connolly surface is the van der Waals surface of a molecule that is accessible to solvent molecules, in this case water. A Connolly surface is generated by rolling a probe sphere over the van der Waals surface of the molecule of interest [26].

The green dotted area in Figure 7a represents the Connolly surface of sulfamerazine, and the structures of sulfanilamide (Figure 7b) and sulfaphenazole (Figure 7c) were superimposed onto the Connolly surface of sulfamerazine. Changes in the sulfonamide R-group produced significant changes in the shape of the sulfonamides tested. Figure 7b shows that reducing the R-group at position 7 in the sulfanilamides resulted in changing the molecular shape compared to that of sulfamerazine. More importantly, the loss of one hydrophobic center (Hy) and one HB acceptor atom (AA) in sulfanilamide may be responsible for important MAb_SMR_ binding interactions. However, increased R-group volume also decreased the binding affinity of sulfonamide analogs to the MAb_SMR_, as shown by sulfaphenazole whose phenazole group protrudes from the Connolly surface of sulfamerzine (Figure 7c), again suggesting that a strict fit between the MAb_SMR_’s binding pocket and the sulfonamide R-group is important. The protruding bulky phenazole group is likely restricted by residues from the MAb_SMR_. It may be concluded that the methyl-pyrimidine of sulfamerazine may be deeply buried in the MAb_SMR_’s binding pocket, while other sulfonamide analog R-groups may not be able to enter the binding pocket as well or the R-group may be too small, resulting in weaker binding interactions.

## 3. Experimental Section

### 3.1. Tracer and Monoclonal Antibody

The tracer used in the binding affinity determinations, fluorescein isothiocyanate (FITC) labelled sulfamethazine (SMZ-FITC), was synthesized and purified using thin-layer chromatography (TLC) in a similar way to the method used to make sulfamerazine-FITC [21]. The MAb_SMR_ was previously raised from mice immunized with sulfamerazine bound to bovine serum albumin using glutaraldehyde as the coupling reagent [21].

### 3.2. Sulfonamides

Sulfamerazine, sulfamethazine, sulfadiazine, sulfadimethoxine sulfameter, sulfathiazole, sulfamethoxypyridazine, sulfamoxole, sulfapyridine, sulfaquinoxaline, sulfachloropyridazine, sulfamethizole, sulfamethoxazole, sulfamonomethoxine, sulfisoxazole, sulfaphenazole and sulfanilamide were purchased from Sigma-Aldrich (St. Louis, MO, USA).

### 3.3. Fluorescence Polarization Immunoassay

The binding affinity (IC_50_, 50% inhibition of control activity) values of the MAb_SMR_ with 17 sulfonamides were determined by a previously developed FPIA method [21].

The ability of the sulfonamides to compete with the tracer SMZ-FITC for binding with the MAb_SMR_ was studied by measuring inhibition curves. The inhibition curves were constructed using a sulfonamide stock solution diluted with borate buffer (50 mM, pH 8.0, 0.1% sodium azide) to give 0.01, 0.1, 1, 10, 100, 1000, 10,000, 100,000 ng/mL. The SMZ-FITC tracer solution (approximately 10× background signal of borate buffer) was prepared by dissolving SMZ-FITC with borate buffer. The assay was conducted as follows: Six hundred and fifty microliters of a sulfonamide standard solution, 650 μL of the tracer solution and 650 μL of the MAb_SMR_ at the optimal dilution (1/34,500) were sequentially added to 3 mL cuvettes, mixed, and following a 5 min incubation at room temperature, the fluorescence polarization was measured. The IC_50_ values were converted to Log_10_IC_50_.

### 3.4. Energy Minimization

Minimum energy conformations of all 17 sulfonamide analogs were calculated using the Minimize module of Sybyl 7.0. The force field was calculated with MMFF94 at an 8 Å cutoff for non-bonded interactions, and the atomic point charges were also calculated with MMFF94. Minimizations were achieved using the consecutive steepest descent method for the first 100 steps, conjugate gradient (Powell) and quasi-Newton (BFGS; named for its originators, approximates the inverse of the Hessian matrix) energy minimization steps until the root-mean-square (RMS) of the gradient became less than 0.005 kcal/mol Å.

### 3.5. Data Set Alignment Using DISCOtech

The DISCOtech program in Sybyl 7.0 was used to align the pharmacophores because it could superimpose conformations reflecting the best binding affinity of the analogs. DISCOtech is based on the assumption that a given set of molecules related by their pharmacological activities may possess consensus features [27]. A stochastic structure conformation search was run to generate a maximum of 100 conformers for each molecule on the basis of maximum diversity to cover as many probable conformers as possible. Since sulfamerazine was used as the hapten to generate the MAb_SMR_, sulfamerazine was selected as the reference compound. For each conformation, the possible pharmacophoric elements were assigned. Five (or fewer) conformations with maximum diversity were selected for each molecule and aligned to sulfamerazine. DISCOtech initially assigns pharmacophore elements such as HB donor atoms, HB acceptor atoms, charged centers, hydrophobic groups, and the most likely location of sulfonamide binding sites with the MAb_SMR_. The distances between the feature points in each sulfonamide conformation were calculated and compared with those of the reference compound. The distance tolerance was set stepwise from 0.25 to 2.5 Å by 0.25 Å increments. If all the intra-molecular distances of identical features between the reference conformation and the calculated conformations of other sulfonamides were met within the tolerance, a valid pharmacophore model was established. The final pharmacophore model with the highest score and lowest pairwise tolerance was proposed and subjected to CoMFA and CoMSIA calculations. The score was calculated according to the following formula [28]:

$$Score=\frac{H}{M}\sum_{allfeatures}\frac{d_{ij}-d_{o}}{k/(k-1)}10^{\left(k-0.5\sum_{alloverlaps}ij\right)}$$

where *H* is the number of molecules that match the model, *M* is the number of targets (molecules in the input set), *k* is the number of features, *d_ij_* is the interfeature distance, and *d_o_* equals 2 Å.

### 3.6. CoMFA Analysis

For CoMFA calculations [27], the alignment molecules were placed in a 3D-cubic lattice with a 2 Å grid in the *x*, *y* and *z* directions. The default *sp*^3^-hybridized carbon atom with a +1 charge was selected as the probe atom for the calculation of the steric (Lennard-Jones 6–12 potential) and electrostatic fields (Coulombic potential) around the aligned molecules with a distance-dependent dielectric constant at all lattice points. Values of steric and electrostatic energy were truncated to 30 kcal/mol avoiding infinity energy values inside the molecules.

### 3.7. CoMSIA Analysis

Similarity indices descriptors were derived with the same lattice box that was used in the CoMFA calculations. The CoMSIA method defines five fields: steric, electrostatic, hydrophobic, HB donor and HB acceptor. A probe atom with a radius of 1.0 Å, +1 charge, hydrophobicity of +1.0, and HB donor and HB acceptor properties of +1.0 was used to calculate steric, electrostatic, hydrophobic, and HB donor and HB acceptor fields. Gaussian type distance dependence was determined between the grid point and each atom of the molecule, and the default value for the attenuation factor (*α*) was set to 0.3 [14,15,29].

### 3.8. Partial Least Squares (PLS) Regression Analysis

PLS methodology was used for all 3D-QSAR analyses to determine the significance of the models. Column filtering was set to 1.0 kcal/mol to speed up the analysis and reduce noise so that only those steric and electrostatic energies with values greater than 1.0 kcal/mol were considered in the PLS analysis. The CoMFA and CoMSIA descriptors served as independent variables and the Log_10_IC_50_ values as dependent variables in PLS regression analyses. The predictive value of the models (*q*^2^) was evaluated using the leave-one-out (LOO) cross-validation method. The cross-validated coefficient, *q*^2^ *_cv_*, was calculated using the following equation:

$${q^{2}}_{cv}=1-\frac{\sum {\left(Y_{pre}-Y_{\text{exp}}\right)}^{2}}{\sum {\left(Y_{\text{exp}}-Y_{mean}\right)}^{2}}$$

where *Y_pre_* represents the calculated binding affinity, *Y_exp_* is the experimentally determined binding affinity, and *Y_mean_* is the mean value of the target property (Log_10_IC_50_).

The optimum number of components used to derive the non-cross-validated model was defined as the number of components leading to the highest non-cross-validated *r*^2^, standard error of the estimate, and F value. At the same time, the CoMFA and CoMSIA color contour maps were derived for the steric and electrostatic fields. The quality of the final CoMFA and CoMSIA models was measured by two statistical parameters: *q*^2^ *_cv_* and *r*^2^. The value of *q*^2^ *_cv_* indicates the predictive capacity of the model, and should be greater than 0.5; and the value of *r*^2^ shows the self-consistency of the model, and should be greater than 0.90 [30].

### 3.9. Connolly Surface

The Connolly surface was calculated for sulfamerazine using the standard implementation in the Sybyl 7.0 package. To calculate the Connolly surface of sulfamerazine, the probe sphere radius was set to 1.4 Å, corresponding to the van der Waals radius of water.

## 4. Conclusions

This paper provides a detailed QSAR that includes CoMFA and CoMSIA studies on 17 sulfonamide analogs binding the MAb_SMR_ produced against sulfamerazine. The pharmacophore model using DISCOtech and the Connolly surface analysis were investigated to show the differences in sulfonamide analogs resulting in different observed FPIA derived binding affinities. DISCOtech revealed that differences in structural size and shape were the primary reasons for the different observed MAb_SMR_ binding affinities. The Connolly surface analysis was a useful tool for comparing molecular structures to a predicted binding surface, and may be useful to help discern factors governing interactions of small molecules and antibodies. The developed CoMFA and CoMSIA models had excellent agreement with 15 of the 17 sulfonamides studied, and will be able to predict binding affinities for the MAb_SMR_ with new sulfonamides. Results from this multidisciplinary research can also provide insights into key structural elements required to design new haptens for development of more desirable antibodies.

## Figures and Tables

**Table 1:** Experimental and Predicted Sulfonamides Binding Affinity to MAb_SMR_.

Drugs a	R	IC_50_	Log_10_IC_50_ (exp.)	CoMFA		CoMSIA	
				Log_10_IC_50_ (pre.)	Residual	Log_10_IC_50_ (pre.)	Residual
SMR	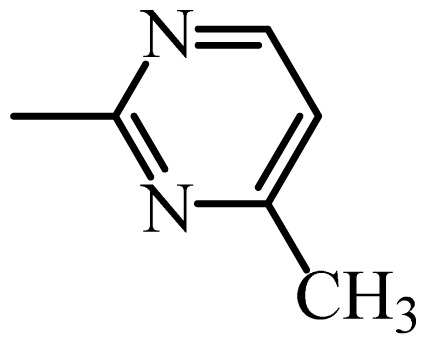	19	1.28	1.24	0.04	1.22	0.06
SMZ	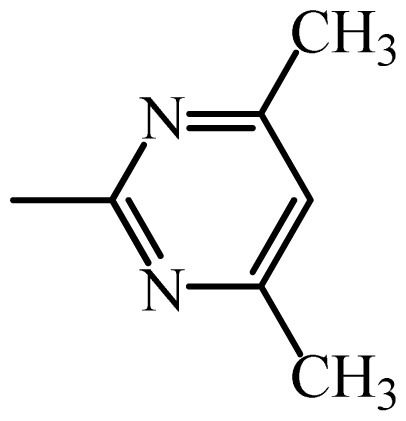	65.7	1.82	1.87	−0.05	1.93	−0.11
SDZ	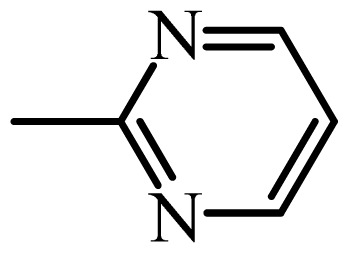	137	2.14	2.10	0.04	2.17	−0.03
SDM	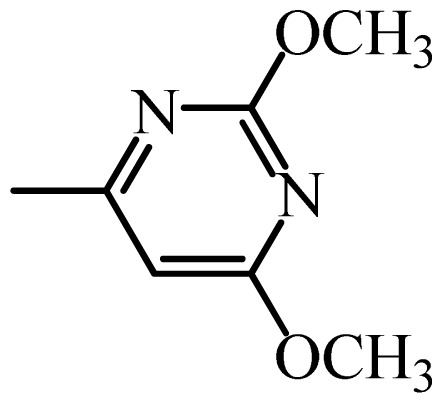	528	2.72	2.77	−0.05	2.75	−0.03
SME	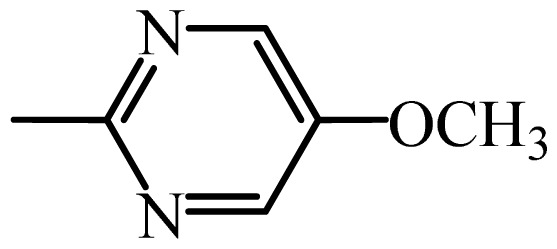	760	2.88	2.87	0.01	2.88	0.00
STZ	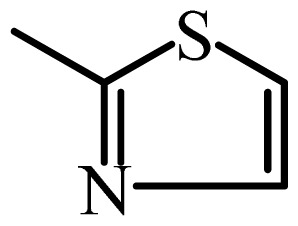	1000	3.00	3.03	−0.03	3.00	0.00
SMP	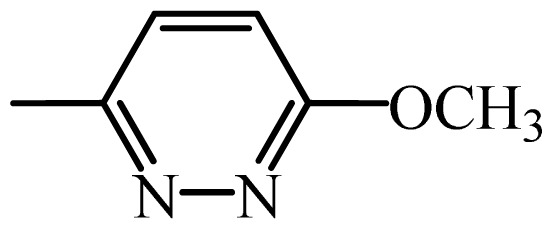	1056	3.02	3.01	0.01	3.02	0.00
SMO	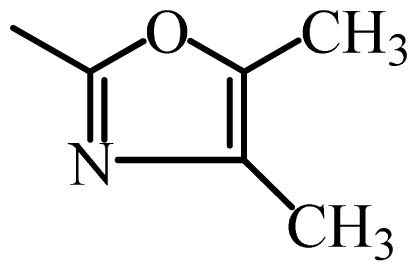	1118	3.05	3.01	0.04	2.89	0.16
SPY	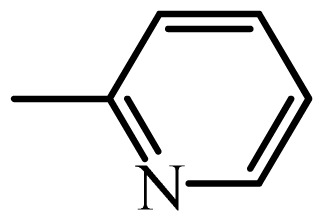	1189	3.07	3.10	−0.03	3.11	−0.04
SQX	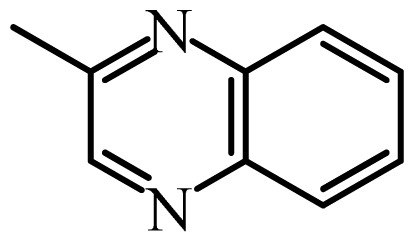	1538	3.20	3.20	0.00	3.17	0.03
SCP	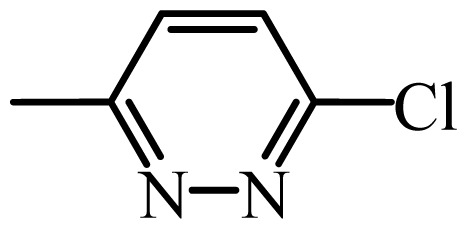	3800	3.58	3.55	0.03	3.60	−0.02
SMT	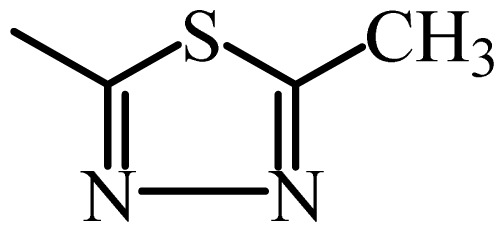	6333	3.80	3.83	−0.03	3.82	−0.02
SMX	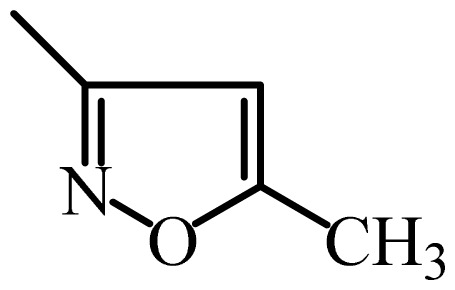	9048	3.96	3.94	0.02	3.88	0.08
SMM	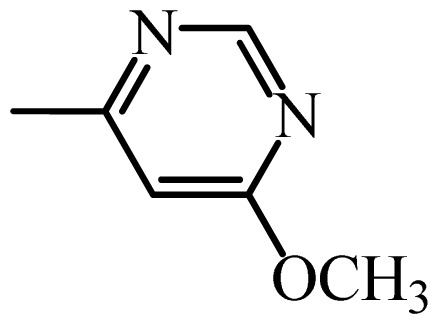	12,667	4.10	4.09	0.01	4.10	0.00
SFX	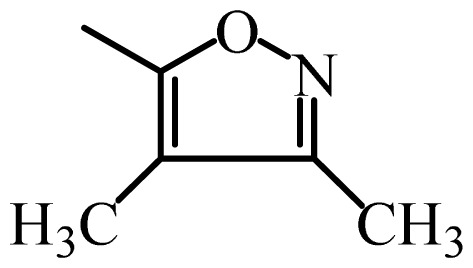	19,000	4.28	4.27	0.01	4.33	−0.05
SPA	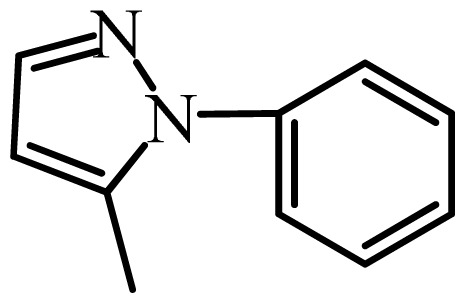	>190,000	-	-	-	-	-
SAM	H	>190,000	-	-	-	-	-

a The sulfonamide abbreviations are as follows: Sulfamerazine (SMR), sulfamethazine (SMZ), sulfadiazine (SDZ), sulfadimethoxine (SDM), sulfameter (SME), sulfathiazole (STZ), sulfamethoxypyridazine (SMP), sulfamoxole (SMO), sulfapyridine (SPY), sulfaquinoxaline (SQX), sulfachloropyridazine (SCP), sulfamethizole (SMT), sulfamethoxazole (SMX), sulfamonomethoxine (SMM), sulfisoxazole (SFX), sulfaphenazole (SPA), and sulfanilamide (SAM).

**Table 2:** Summary of Results of CoMFA Analysis.

				No validation		
				
Models	Delete compounds	Leave-one-out (LOO) *q*^2^	Cross-validated *q*^2^ *_cv_*	Standard error of the estimate (SEE)	*r*^2^	*F* value
M1	-	0.258	0.241	-	-	-
M2	SAM	0.126	0.127	-	-	-
M3	SPA	0.310	0.234	-	-	-
M4	SAM, SPA	0.582	0.600	0.071	0.995	397.263

*q*^2^, LOO correlation coefficient; *q*^2^ *_cv_*, cross-validated correlation coefficient; *r*^2^, non-cross-validated correlation coefficient; *F* value, *F*-statistic for the analysis.

**Table 3:** Summary of Results of CoMSIA Analysis.

Models	Steric and electrostatic	Hydrophobic	Donor and acceptor	Leaveone- out (LOO) *q*^2^	Crossvalidated *q*^2^ *_cv_*	SEE	*r*^2^	*F* value
N1	√	-	-	0.558	0.678	-	-	-
N2	-	√	-	−0.491	−0.593	-	-	-
N3	-	-	√	0.407	0.431	-	-	-
N4	√	√	√	0.258	0.287	-	-	-
N5	√	-	√	0.450	0.523	0.078	0.994	324.629
N6	√	√	-	0.145	0.133	-	-	-
N7	-	√	√	0.084	0.021	-	-	-

*q*^2^, LOO correlation coefficient; *q*^2^_cv_, cross-validated correlation coefficient; SEE, standard error of the estimate; *r*^2^, non-cross-validated correlation coefficient; *F* value, F-statistic for the analysis.
